# HPAIV outbreak triggers short-term colony connectivity in a seabird metapopulation

**DOI:** 10.1038/s41598-024-53550-x

**Published:** 2024-02-07

**Authors:** Jana W. E. Jeglinski, Jude V. Lane, Steven C. Votier, Robert W. Furness, Keith C. Hamer, Dominic J. McCafferty, Ruedi G. Nager, Maggie Sheddan, Sarah Wanless, Jason Matthiopoulos

**Affiliations:** 1 School of Biodiversity, One Health and Veterinary Medicine, University of Glasgow, Glasgow, UK; 2 RSPB Centre for Conservation Science, Sandy, UK; 3 School of Energy, Geoscience, Infrastructure and Society, The Lyell Centre, Herriot Watt University, Edinburgh, UK; 4 MacArthur Green, Glasgow, UK; 5 University of Leeds, Leeds, UK; 6 Scottish Seabird Centre, North Berwick, UK; 7 UK Centre for Hydrology & Ecology Edinburgh, Penicuik, UK

**Keywords:** Behavioural ecology, Ecological epidemiology, Animal behaviour

## Abstract

Disease outbreaks can drastically disturb the environment of surviving animals, but the behavioural, ecological, and epidemiological consequences of disease-driven disturbance are poorly understood. Here, we show that an outbreak of High Pathogenicity Avian Influenza Virus (HPAIV) coincided with unprecedented short-term behavioural changes in Northern gannets (*Morus bassanus*). Breeding gannets show characteristically strong fidelity to their nest sites and foraging areas (2015–2019; n = 120), but during the 2022 HPAIV outbreak, GPS-tagged gannets instigated long-distance movements beyond well-documented previous ranges and the first ever recorded visits of GPS-tagged adults to other gannet breeding colonies. Our findings suggest that the HPAIV outbreak triggered changes in space use patterns of exposed individuals that amplified the epidemiological connectivity among colonies and may generate super-spreader events that accelerate disease transmission across the metapopulation. Such self-propagating transmission from and towards high density animal aggregations may explain the unexpectedly rapid pan-European spread of HPAIV in the gannet.

## Introduction

Disease outbreaks can have drastic impacts on wild animal populations, particularly in colonial animals such as seals and seabirds that congregate in high densities at breeding sites. For example, phocine distemper outbreaks resulted in mass mortality in the Northern European harbour seal (*Phoca vitulina*) population in 1988 and 2002, respectively^1^. In 2021, High Pathogenicity Avian influenza (HPAI H5N1) spilled over into European seabirds for the first time and by 2022 the outbreak had caused mass mortality on a global scale, particularly in great skuas (*Stercocarius skua*), sandwich terns (*Thalasseus sandvicensis)*^2,3^ and Northern gannets (gannet hereafter, *Morus bassanus*)^4^. However, the mechanisms underpinning the observed rapid spread of this novel threat to seabirds are unknown.

Mass mortality severely disturbs the breeding environment of surviving animals, but the behavioural consequences of disease-driven disturbance and the resulting ecological and epidemiological implications are poorly understood, since monitoring fine-scale animal behaviour and movement rarely coincides with such unpredictable events. However, animals can modify their behaviour and movements in response to anthropogenic disturbances^5^, as evidenced by changes in space use patterns or shifts to nocturnality in response to human disturbances^6,7^. Such modifications can also have pronounced consequences for the transmission of diseases. For example, culling, as an effort to control disease, has instead promoted pathogen spatial spread in vampire bats, *Desmodus rotundus*, and badgers, *Meles meles*, since the disturbance associated with culling increased dispersal^8,9^. However, questions remain over the possibility for positive feedbacks between infection, mobility and subsequent increased transmissivity.

Here, we investigated the impact of an HPAIV outbreak on the movement behaviour of adult gannets before, during and shortly after the epidemic at Bass Rock, UK, the world’s largest gannet colony with ~ 75,000 breeding pairs^10^. The movement ecology of adult breeding gannets is exceptionally well studied with satellite and GPS tracking studies from 1998 to the present^11–24^, providing a data-rich baseline for comparison. Adult gannets are highly site-faithful, returning each year to the same colony and, mostly, the same nest site^25^. If a breeding attempt fails, birds continue nest site ownership^14^ and maintain a regular routine of commuting between colony and foraging sites at sea^14^. Incubating and chick-rearing birds display highly predictable movements to individual-specific foraging areas^14,24^ and forage in colony-specific, non-overlapping ranges during breeding^11^. Taken together, these behavioural and space use characteristics suggest limited potential for contact among breeding colonies, making adult gannets unlikely candidates for disease transmission across the metapopulation.

**Figure 1:**
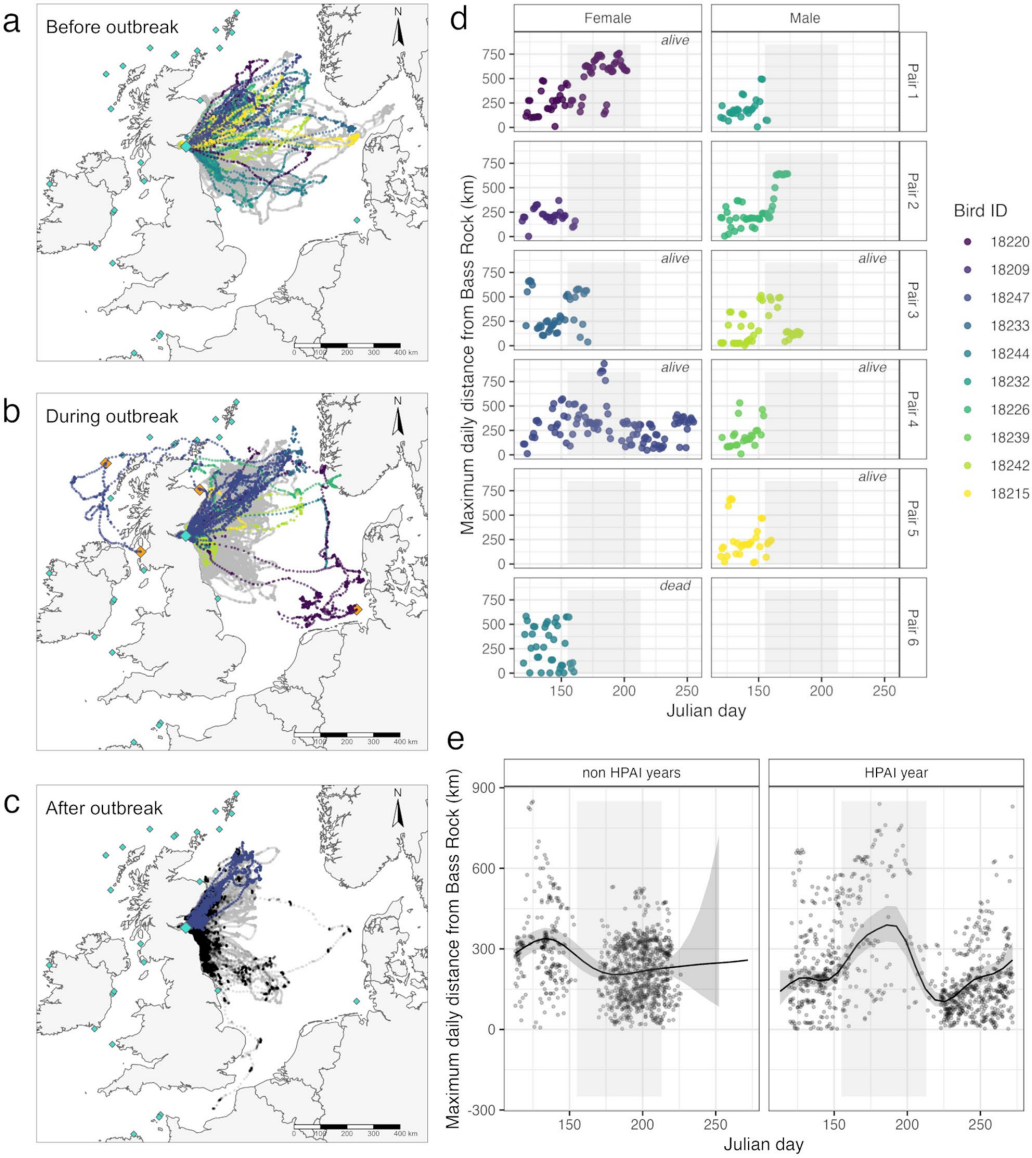
(**a**) Movements of breeding adult gannets before the outbreak of HPAIV on the Bass Rock before the 4th of June, (**b**) during the outbreak up until 31st July and (**c**) after the outbreak. Gannets GPS-tracked from April 2022 onwards are individually coloured (see legend), positions for gannets tagged in 2015–2019 in grey and from August 2022 in black. The study colony Bass Rock is indicated by a large turquoise diamond, colonies visited by GPS tracked birds are shown as large orange diamonds and unvisited colonies are shown as small turquoise diamonds. The background map was created in R 4.3.2, based on the freely accessible 1:10 m world coastlines shapefile downloaded from https://www.naturalearthdata.com/downloads/10m-physical-vectors/ (**d**) Maximum daily distances from the Bass Rock colony for gannet pairs tracked during the HPAI outbreak, individual colours correspond with movement data in A, B and C. (**e**) The relationship between maximum daily distance and Julian day for all other years (left) and the HPAI year (right, gamm prediction, confidence interval and raw data). The light shaded area in (**d**) and (**e**) refers to the time period during the HPAIV outbreak corresponding to the map in (**b**).

We deployed 18 g nanofix GPS-GSM devices (Pathtrack Ltd.) with TESA tape to the tail of ten gannets from six breeding pairs in April 2022, before egg-laying, and confirmed that each pair incubated an egg in late May 2022. On the 4 June 2022, the first clinical symptoms of HPAIV were observed in gannets on the Bass Rock and an outbreak of clade 2.3.4.4b HPAIV H5N1 was confirmed shortly thereafter^4^. Over the following month, the outbreak severely reduced the colony size and suppressed adult apparent survival to 0.455%^4^. All study birds had lost their egg/chick by 15 June 2022. Six birds survived the outbreak, one bird was found dead and the fate of the remaining three birds is unknown (Fig. 1d, Table S1). To investigate the behaviour of breeding gannets following the HPAIV outbreak, we also deployed nanofix GPS GSM tags to ten breeding gannets with chicks captured in mid-August 2022, approximately 2.5 months following the onset of the HPAIV outbreak on the Bass Rock.

## Results and discussion

Of the ten birds tracked from April 2022, five birds kept transmitting during the HPAIV outbreak for more than one week following the onset of the outbreak (Fig. 1d, Table S1). Of these, three performed unprecedented long-distance movements away from the Bass Rock, to novel locations outside the geographic range of the regular movements of gannets tracked in previous years and earlier in the season prior to the HPAI outbreak (Fig. 1a–c, supplementary material gifs for pair 1, pair 2 and pair 4). The movement patterns of gannets in the HPAI year therefore differed significantly from the movement patterns of 120 incubating and chick rearing gannets GPS tagged in 2015–2019 at Bass Rock (Fig. 1b,c). In the years prior to 2022, maximum travel distances from the colony peaked in late May during the incubation period, when breeding adults are not yet constrained by a dependent chick, were significantly lower during the early chick-rearing period (early July, from julian day 175) and plateaued at similar distances as chick rearing progressed (Fig. 1c, e, Julian day: non-HPAIV year, edf = 4.378, F = 12.32, *p* < 0.01, mixed effects generalised additive model). In contrast, during the HPAIV outbreak in 2022, maximum travel distances increased drastically throughout June, peaked in mid-July around julian day 175 to maximum daily distances of around 100 km beyond those reached in previous years, before decreasing around 2.5 months after the onset of the outbreak (Fig. 1e, julian day:HPAI year, edf = 8.35, F = 29.19, *p* < 0.01). A slight increase in maximum daily distances towards the end of the data series was driven by a small number of locations likely associated with a longer trip offshore and the onset of migration of one of the study birds in late September, respectively (Fig. 1c,e).

During the unusual long-distance movements during the HPAIV outbreak, two GPS-tracked gannets visited other gannet breeding colonies (Fig. 1b, supplementary material Figure S1–S5) and a third bird visited freshwater lakes ~ 50 km inland in Norway before transmission ceased (Fig. 1b, SM Figure S6–S7). None of the 120 birds tracked on the Bass Rock before 2022, nor any of the hundreds of breeding gannets tracked from 16 colonies over more than two decades^11,14–16,21,23,24,26,27^, nor any tracked failed breeder^14^ has been recorded visiting a different gannet breeding colony or inland area. Prospecting different colonies provides colonial animals with information on breeding site quality, which can inform recruitment^28^, but in seabirds, prospecting is primarily associated with immature seabirds^29^. Breeding failure may initiate prospecting in some instances, although the evidence in seabirds points primarily to members of the Laridae which tend to have lower philopatry than other seabird taxa^30^. In contrast, breeding gannets are highly faithful to their breeding colony, acquiring their breeding sites during a prolonged, high-investment process over several years^25^. Previous tracking of failed breeders revealed that they are less constrained than breeders but did not show the long-distance and inter-colony movements described here^14^, suggesting that breeding failure alone did not trigger these drastic changes in space use patterns. Food-weather interactions and predation have previously been shown to lead to colony abandonment in arctic terns, *Sterna paradisaea*^31^. There is no evidence available for Northern gannets however, closely related Cape gannets, *Morus capensis*, maintain nest sites and foraging ranges under extreme food stress and years of breeding failure^32^. It appears that the outbreak of a highly lethal disease and the resulting severe and obvious decay in the quality of the breeding habitat surrounding the surviving birds may be a sufficiently drastic disturbance that has the potential to decrease the high site faithfulness of gannets. Why do gannets respond in such a way? The current evidence does not allow us to venture beyond speculation, but if these behavioural changes represent an adaptive response to disease avoidance, it is certainly one that is not observed in response to other forms of disturbance such as marine heatwaves or extreme weather events.

The drastic changes in space use patterns we describe here appear to be a short-term response to the HPAIV outbreak, albeit at the peak infectious period, rather than a more permanent modification: breeding gannets with surviving chicks that were GPS-tracked several months after the disease outbreak maintained their well-established routines and did not visit different breeding colonies (Fig. 1c), but foraging distances and trip durations were shorter than these of breeding birds tracked in a previous year (see also Fig. 1c,e), possibly in response to the reduced density of competing conspecifics^33^.

Long-distance movements are only relevant for disease transmission if the moving animal is infectious at the time of movement. The limited evidence currently available on the effect of HPAIV infection on movement is ambiguous: a recent study on griffon vultures, *Gyps vulvus*, shows that infected individuals were more lethargic or even immobile thus probably reducing the reducing transmission risks^34^ while movements of infected mallards, *Anas platyrhynchos*, did not differ from unaffected individuals whilst sharing space with the latter, suggesting that movements of infected birds contributed to the maintenance and dispersal of HPAI^35^. We do not have information on the disease status for our GPS-tracked animals since they were captured well before the disease outbreak. The study animals that we tracked during the HPAIV outbreak were most likely exposed to the virus since they nested in an area where in-situ observations showed severe mortality of adult breeders^4^. Indeed, one of our study birds that had normal eyes upon capture in April and that performed long-distance movements during the outbreak was resighted late in the season with one black eye, a phenotypic signal of a previous HPAIV infection^4^. The duration of viral shedding for HPAIV for seabirds is unknown, but data from six experimentally infected wild duck species suggests that live virus can be shed from 1 to 2 days after inoculation, for periods between 5 and 14 days^36^, hence the onset and duration of long-distance movements described here fall well within such a timeframe.

Considering the modest sample size of GPS-tagged animals during the HPAIV outbreak the proportion of survivors that performed unprecedented long-distance movements is astonishingly high. If the pattern scales up to the colony level, the outbreak of HPAIV may self-propagate transmission by altering gannet movement and aggravating the connectivity between different gannet colonies as well as causing incursions into novel habitat types with susceptible animal communities. Such super-spreading^37^, even by a small number of individuals, might explain the rapid spread through the gannet colony networks in the West and East Atlantic^4^. HPAI is a novel, fundamental and poorly understood threat to seabird metapopulations. We recommend focussing future research efforts in seabirds on linking studies on movement ecology with sample collection to infer the disease status of the study animals and on modelling HPAIV spread in seabird metapopulations to close the knowledge gap on mechanisms of HPAIV transmission dynamics.

## Material and methods

### *Permits* *and ethical approval*

All our methods were carried out adhering to relevant guidelines and regulations and are reported in accordance with ARRIVE guidelines. The fieldwork was performed in partnership with the Scottish Seabird Centre with permission of the landowner of the Bass Rock, Sir Hew Dalrymple, and under a special methods endorsement and ringing permit of the British Trust for Ornithology BTO to Jana Jeglinski. Colour ringing was performed under a permit to Jude Lane from the BTO. We conducted our fieldwork under an exemption to the general ringing ban in seabird colonies granted by NatureScot which permitted us to capture and tag gannets during the HPAIV outbreak. All fieldwork was performed with the required risk assessments and ethical approval issued by the ethics committee of the university of Glasgow and the BTO in place.

### GPS tracking—2022 (HPAI year)

We captured five breeding pairs of gannets on 29th and 30th April 2022 at Bass Rock, UK (56° 04′, 2° 29′), about one month before the HPAIV outbreak at this colony. Pairs on nest sites were caught before egg-laying (Jeglinski et al., in prep.). To provide insights into the behaviour of gannets following the HPAIV outbreak, we also captured 10 breeding adults with 2–8 weeks old chicks between the 11th and 14th August 2022, about 2.5 months after the start of the HPAIV outbreak on the Bass Rock. A detailed analysis of the August dataset is described in^33^. 

For the captures, we used a 6 m telescopic pole with a hook or noose attached to the end and restrained the birds in a custom-designed ‘gannet-jacket’ that allows efficient handling and weighing to minimize capture stress for the bird. We weighed birds with a digital scale (Kern, precision 0.1 kg), and took morphometric measurements with a metal ruler (precision 1 cm) and a digital calliper (precision 0.01 mm). As far as possible, we targeted previously colour-ringed birds with known successful breeding status in previous years, and ringed unmarked birds with a BTO metal ring and a blue–white high impact acrylic colour ring with a unique alphanumeric combination. Birds were assumed to form a pair because they performed courtship behaviours and attended the same nest site. All pairs consisted of at least one previously marked individual so that the sex of each bird was known or inferred based on the sex of its partner. We attached nanofix GPS-GSM tags (Pathtrack Ltd., Otley, UK, 18 g) to the central three tail feather using TESA 4651 tape. Tags were programmed to record the birds’ location every 15 min and scheduled to send 12-hourly data packages via the mobile phone network to a server, from where we accessed data in near real-time. The tags are solar powered and have a built-in dynamic algorithm that adjusts the GPS fix frequency depending on battery charge.

The total handling time lasted ~ 10 min. Once released, most birds returned immediately to their nests. We monitored all birds after capture and on subsequent trips to verify pair continuity and nest status. We confirmed in late May that all pairs tagged in April were incubating an egg. One pair had split up or been erroneously captured as a pair, but both birds were incubating an egg with unmarked partners. Access to the colony following the August captures was restricted due to the HPAIV outbreak, but we resighted one GPS tagged bird captured in August with a chick in late September—the others were not resighted, but chicks might have already fledged at this late stage of the breeding season.

### GPS tracking—non HPAI years

For comparison with the data from the HPAI year 2022, we used GPS tracking data for incubating or breeding Bass Rock gannets collected during 2015–2019 (150 deployments; n = 120 individual birds). Briefly, the capture methodology was very similar to the approach we took in 2022, but instead of GPS-GSM tags, small GPS loggers (igotU-GT600, Mobile Action Technology, Taipei, Taiwan, 33 g) with a fixed sampling interval of 2 min were deployed, both before incubation in late April (2017–2019) and during chick rearing from mid-July (2015–2019). Tags deployed on birds during April were retrieved mainly in June, tags deployed during chick-rearing were retrieved after 7–14 days^27^.

### Maximum daily distance from capture colony

Prior to any spatial data analyses, we transformed all spatial data into the Lambert Azimuth Equal Area LAEA (EPSG code 3035) projection. We curtailed GPS data to the 30th September 2022 to omit GPS locations associated with the onset of migration.

We calculated the maximum daily distance from the Bass Rock breeding colony as a measure of the extent of the space used by each bird. First, using the function *gridDistance* (R package raster^38^), we generated a distance raster with a resolution of 1 km that originated at the Bass Rock and that excluded land thus reflecting biologically realistic travel distances^39^. Overlaying the GPS locations on the distance raster, we extracted the distance (measured in km) of each GPS location to the Bass Rock using the function *st_extract* (R package stars^40^). We calculated the ‘Julian day’ of each GPS location using the function *yday* (R package lubridate^41^) and used the maximum distance for each day, for each bird in each year as response variable for our model. The GPS tracking data pre-2022 was available as foraging trips (containing only locations > 500 m to the Bass Rock for longer consecutive periods than 40 min) while the 2022 data was available in unfiltered form. For consistency with the distance threshold used to identify colony visits (see below), we therefore filtered all data to omit maximum distances to the Bass Rock <  = 2 km prior to statistical analysis (reducing the dataset by 1%).

### Colony visits

For each GPS location, we calculated the nearest distance to any known gannet breeding colony using the *st_nn* function (R package nngeo^42^). Since gannets congregate in ‘rafts’ in the vicinity of colonies and the majority of rafting events were concentrated within a 2 km radius around a colony^43^, we defined a ‘colony visit’ as a visit of more than 15 min (two subsequent GPS locations for 2022 and seven for previous years when sampling rate was higher) within a 2 km radius of a gannet breeding colony. We retained the identity of any visited colony and calculated the duration of the colony visit as the difference between the earliest timestamp of a GPS location within the 2 km radius and the first timestamp of the subsequent location outside of it.

### Statistical analysis

We fitted a mixed-effects generalised additive model using the function *gam* (R package mgcv^44^, Gaussian likelihood, log link) to the data to quantify potential non-linear variation in the relationship between ‘maximum daily distance’ (response variable) and Julian day (explanatory variable). For our full model, we fitted an interaction between smoothed Julian-day and the factor ‘HPAIV status’, classifying all years prior to 2022 as ‘non-HPAI’ (n = 120 individuals) and the year 2022 as “HPAI” (n = 20 individuals, ten gannets tagged in April 2022, ten gannets tagged in August 2022). We accounted for the non-independence of repeated data points from the same bird by fitting a random effect for Bird ID on the intercept. We simplified the full model by removing the interaction term, and by fitting an intercept only model and compared the three candidate models using AIC to select the best model. The best model was the full model with a ΔAIC = 221.38 below the next lowest AIC of the simplified models.

We used R^45^ version 4.3.2 for all data processing and analyses. Code to run the analyses and to generate the figures in this manuscript is supplied in the GitHub repository https://github.com/JanaJeglinskiR/gannet_movements_HPAIV.

### Supplementary Information

Supplementary Information 1.

Supplementary Video 1.

Supplementary Video 2.

Supplementary Video 3.

Supplementary Video 4.

Supplementary Video 5.

Supplementary Video 6.

## Data Availability

The data that support the findings of this study are available from the Movebank repository (Reference Number 2658220054 and 2658117564) and the BirdLife International Seabird Tracking database but restrictions apply to the availability of these data, which were used under license for the current study, and so are not publicly available. Data are however available from the authors upon reasonable request and with permission of Dr Jeglinski, Dr Lane and Dr Hamer.
