# Supplementary material for: HPAIV outbreak triggers short-term colony connectivity in a seabird metapopulation
Source: Sci Rep. 2024 Feb 7;14:3126. doi: 10.1038/s41598-024-53550-x (PMC10850054; doi:10.1038/s41598-024-53550-x)
Supplement: Supplementary file 1 — Supplementary Information 1. [file 41598_2024_53550_MOESM1_ESM.pdf]

Supplementary material: HPAIV outbreak triggers short-term colony connectivity in a seabird metapopulation

Jana W.E. Jeglinski, Jude Lane, Steven C. Votier, Robert W. Furness, Keith C. Hamer, Dominic McCafferty, Ruedi Nager, Maggie Sheddan, Sarah Wanless and Jason Matthiopoulos

Detailed description of long-distance moving birds during HPAIV outbreak

Two birds prospected alternative gannet breeding colonies shortly after the HPAIV outbreak on the Bass Rock. Both prospecting birds were females.

Tag ID 18220

Tag ID 18220 moved to the coast of Norway on the 19th of June, returned to the Bass Rock, and then moved south and visited the colony of Helgoland on the 15th of July 2022 between 07:43 AM and 09:05 AM for a duration of 81 minutes, with the closest distance to the colony centroid recorded as 344 m (Figure S1). Figure S1 illustrates that the bird spent considerable time in the vicinity to Heligoland at sea, within the foraging range of Heligoland gannets [1]

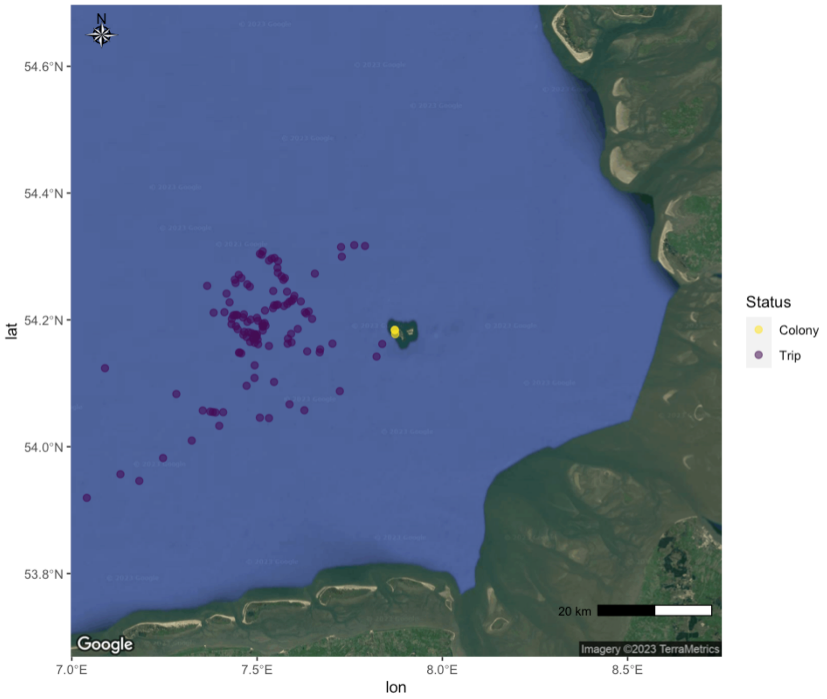

Figure S1: GPS tracking data for Tag ID 18220 illustrating the prospecting visit at the gannet breeding colony Heligoland, Germany. The maps in all supplementary figures were created in R

4.3.2, using a google maps satellite image accessed with an API key through the package *ggmap*[2] as background.

Tag ID 18247

Tag ID 18247 visited three different breeding colonies. On the 28th of June 2022, 18247 visited the Troup Head colony, the nearest neighbouring colony to the Bass Rock, between 4:38 AM and 5:23 AM for a duration of 45 minutes, with the nearest distance to the colony centroid recorded as 1103 m (Figure S2).

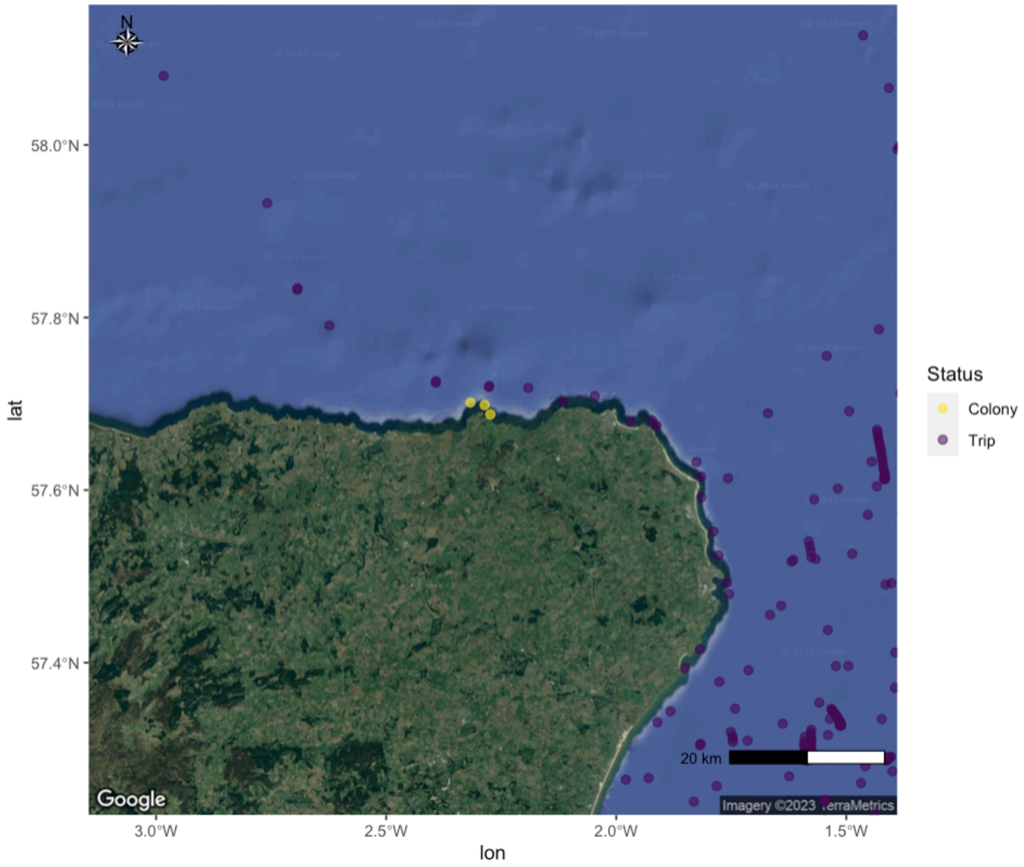

Figure S2: GPS tracking data for Tag ID 18247 illustrating the prospecting visit at the gannet breeding colony Troup Head, Scotland.

Tag ID 18247 then flew past the colony St. Kilda on the 30th of June with one GPS location in a distance of 1122 m which did not qualify as colony visit according to or criteria (see materials and

methods). Tag 18247 returned to St. Kilda on the 2 July 2023 and stayed within 2 km of the colony for 75 minutes, with the closest distance to the colony centroid of 1040 m (Figure S3).

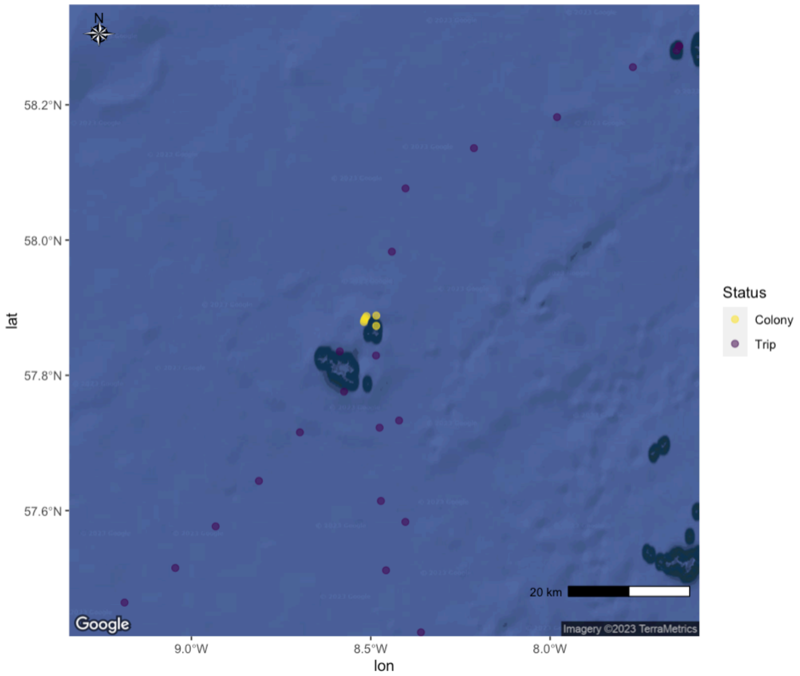

Figure S3: GPS tracking data for Tag ID 18247 illustrating the prospecting visit at the gannet breeding colony St. Kilda, Scotland.

On the 3rd July 2022, 18247 visited the colony Ailsa Craig for a duration of 60 minutes, with the closest distance to the colony centroid measuring 463 m (Figure S4).

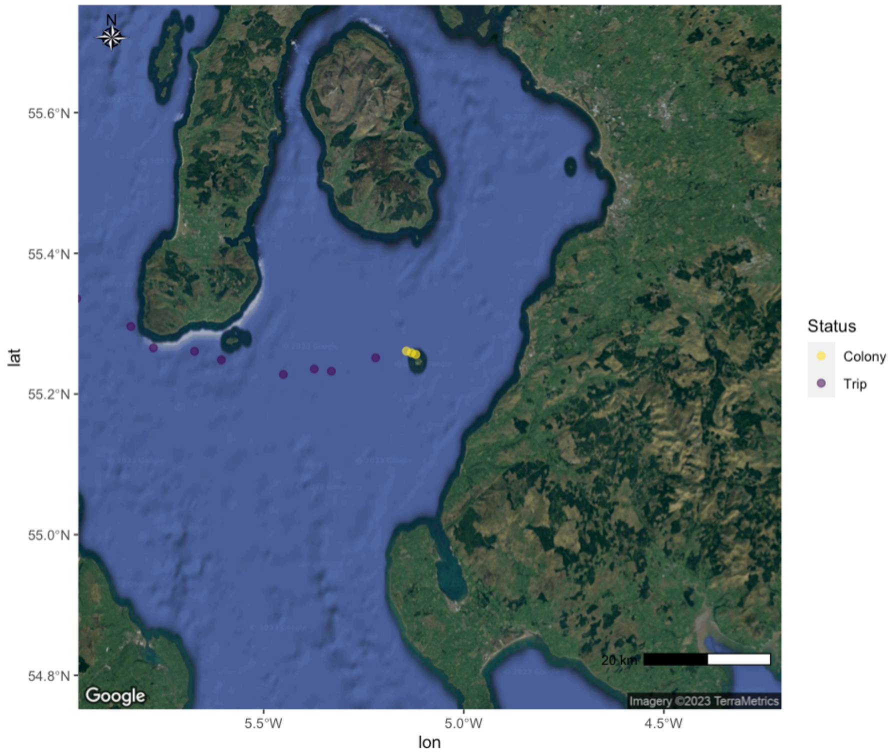

Figure S4: GPS tracking data for Tag ID 18247 illustrating the prospecting visit at the gannet breeding colony Ailsa Craig, Scotland.

18247 also logged one GPS location in a distance of 649 m to the breeding colony Sule Stack (Figure S5) but this did not qualify as colony visit according to our criteria (see materials and methods).

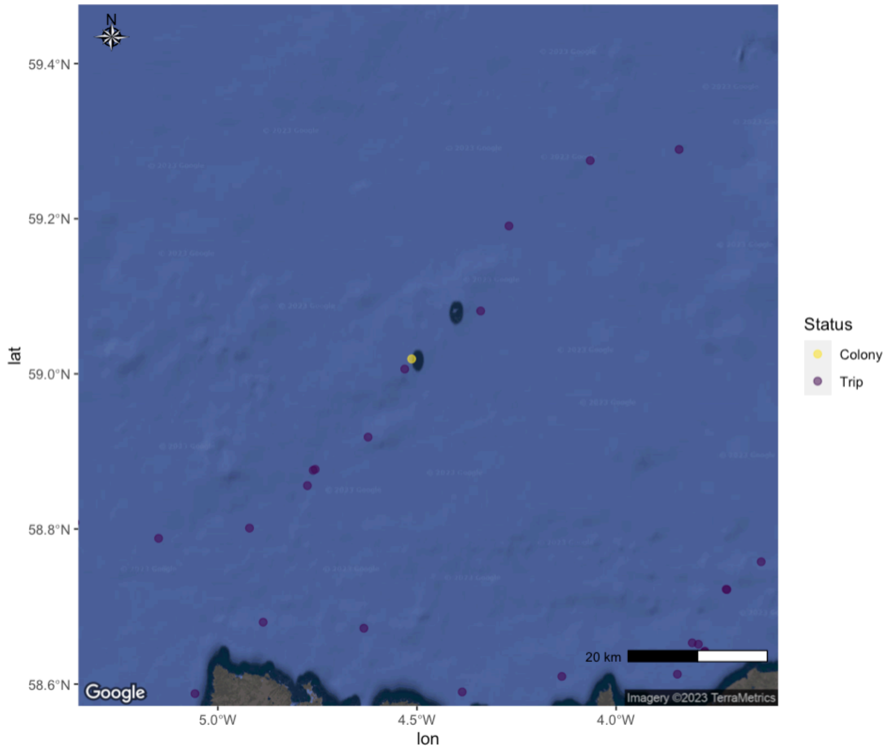

Figure S5: GPS tracking data for Tag ID 18247 illustrating a fly-by at the gannet breeding colony Sule Stack and neighbouring Sula Sgeir, Scotland.

Tag ID 18226

Tag ID 18226, a male gannet, departed from the Bass Rock and flew north towards Wick and the southern Orkney Islands. From there, it flew in a straight path across the North Sea east to the Norwegian coast close to Egersund (Figure S6). The bird first flew approximately 50 km inland, along the lake Ørsdalsvatnet and onwards to come to stay at several smaller waterbodies between

Storvatnet and Stakken (Figure S7), before returning to the coast between Egersund and Sogndalstrand. The signal ceased on the 22 June 2022 at around noon. To our knowledge, this is the first evidence of a GPS tracked gannet moving inland and spending time at an inland waterbody.

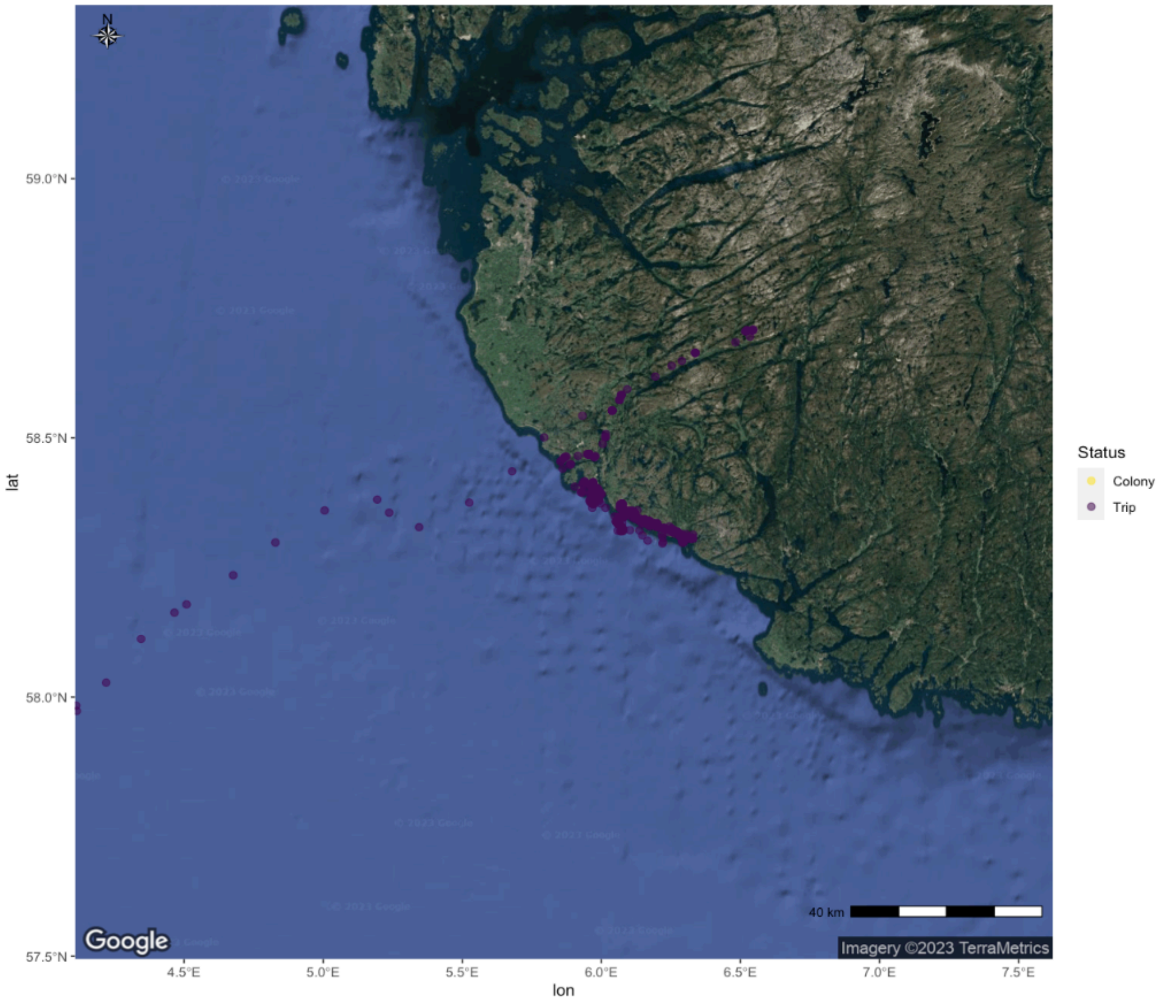

Figure S6: GPS tracking data illustrating inland movements of Tag ID 18226.

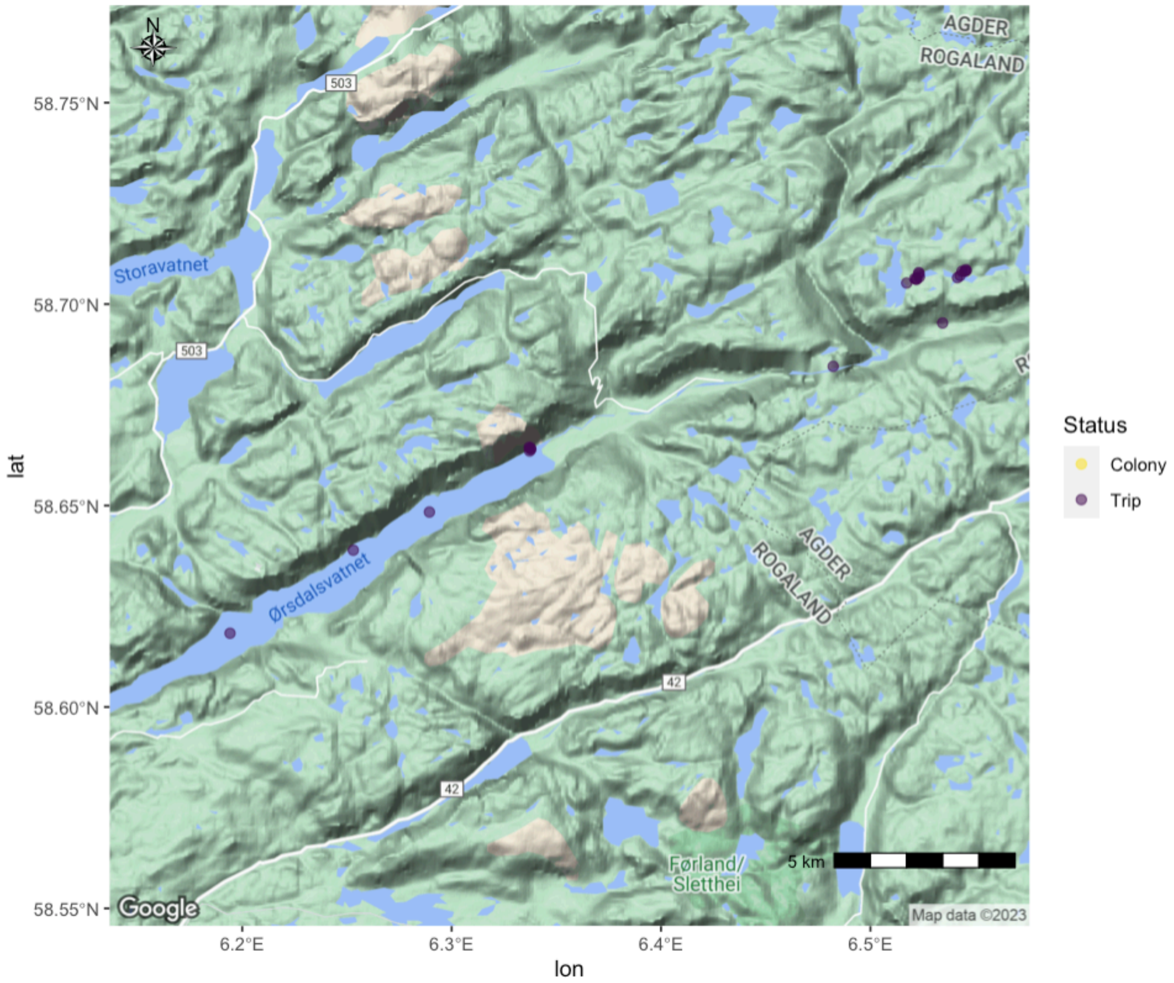

Figure S7: GPS tracking data illustrating the use of inland waterbodies by Tag ID 18226.

Listless gannets have been sighted at unusual locations, e.g. road sides in coastal towns, caravan parcs and inland farm fields during the HPAI outbreak in 2022 and in early 2023 – these observations were mainly shared on Twitter. These birds were assumed to be infected with HPAI and in some cases, confirmed dead shortly after their sighting. Tag ID 18226 moved in a very small area along the coast for about 5 days before the GPS transmission ceased (supplementary material gif pair 2). Without further evidence it is not possible to ascertain the disease status of the bird and if it died, but the unusual geographic location, behaviour and limited mobility appear similar to the sightings of these moribund birds. The maps in all supplementary figures were created in R 4.3.2, using a google maps terrain image accessed with an API key through the package *ggmap*[2] as background.

References

1. Peschko, V., Mendel, B., Mercker, M., Dierschke, J. & Garthe, S. Northern gannets (Morus bassanus) are strongly affected by operating offshore wind farms during the breeding season. *J Environ Manage* **279**, 111509 (2021).

2. Kahle, D. & Wickham, H. ggmap: Spatial visualization with ggplot2. *The R Journal* **5**, 144–161 (2013).

Table S 1: Reference data for the 10 GPS tagged gannets captured in April 2022 and tagged during the HPAI outbreak in June 2022. The outbreak on the Bass Rock commenced on the 4th of June (julian day 155).

ID	Pair ID	GPS tracking duration (days)	Days tracked before outbreak	Days tracked after first day of outbreak	Date transmission date	Status	Remark
18232	1	37	35	2	157	unknown	
18220	1	80	33	47	202	alive	resighted on the Bass Rock without tag 23.09.2022, right eye black, left eye normal
18226	2	53	35	18	173	unknown	
18209	2	42	36	6	161	unknown	
18242	3	64	36	28	183	alive	resighted on the Bass Rock without GPS tag 28.04.2023
18233	3	50	34	16	171	alive	Resighted on the Bass Rock without GPS tag 01.08.2022
18239	4	34	35	-1	154	alive	resighted on the Bass Rock without GPS tag 28.04.2023
18247	4	136	35	101	256	alive	resighted on the Bass Rock without GPS tag 24.09.2022
18215	5	40	35	5	160	alive	resighted on the Bass Rock without GPS tag 28.04.2023
18244	6	40	35	5	160	dead	found decomposed on Stora Varholmen, province Vestra Goetaland 21.07.2022
