# Supplementary figures and images for: HPAIV outbreak triggers short-term colony connectivity in a seabird metapopulation
Source: Sci Rep. 2024 Feb 7;14:3126. doi: 10.1038/s41598-024-53550-x (PMC10850054; doi:10.1038/s41598-024-53550-x)

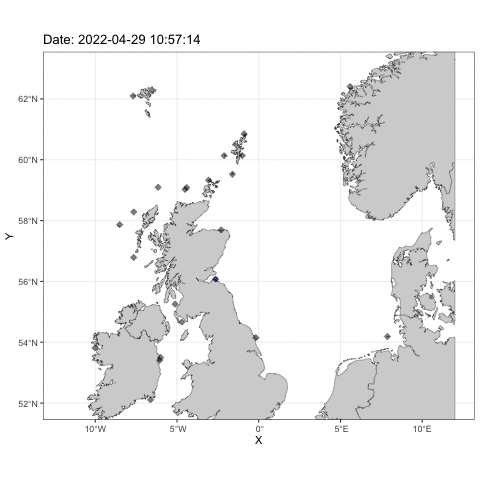

Supplement: Supplementary file 2 — Supplementary Video 1. [file 41598_2024_53550_MOESM2_ESM.gif]

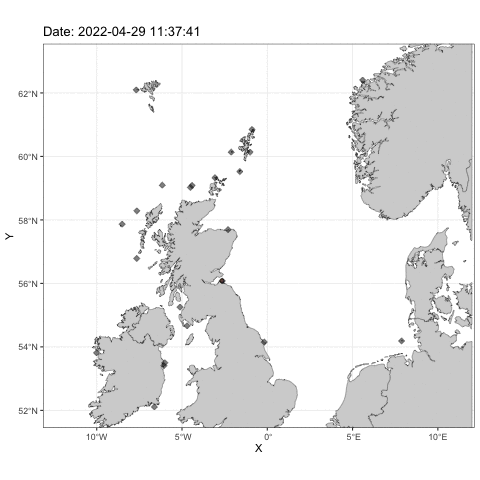

Supplement: Supplementary file 3 — Supplementary Video 2. [file 41598_2024_53550_MOESM3_ESM.gif]

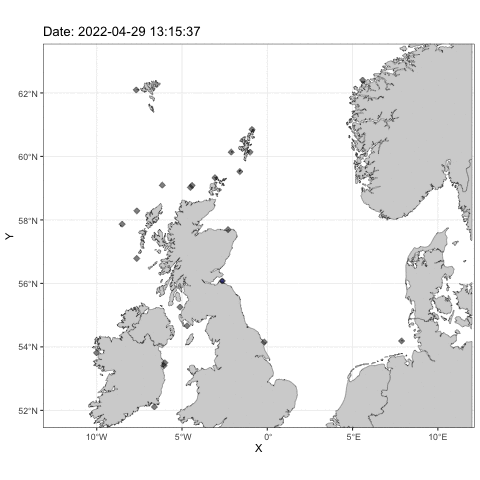

Supplement: Supplementary file 4 — Supplementary Video 3. [file 41598_2024_53550_MOESM4_ESM.gif]

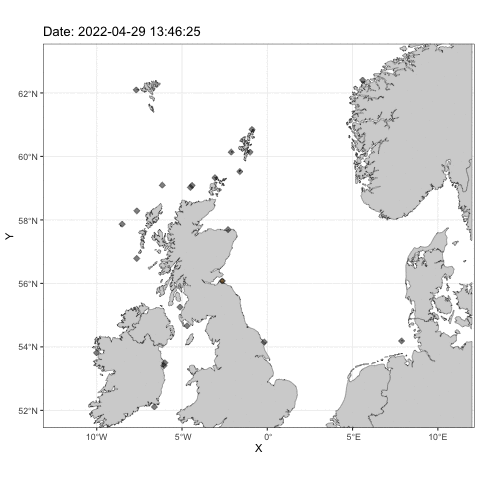

Supplement: Supplementary file 5 — Supplementary Video 4. [file 41598_2024_53550_MOESM5_ESM.gif]

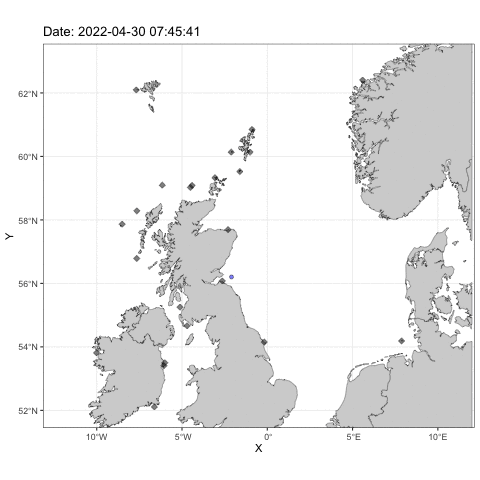

Supplement: Supplementary file 6 — Supplementary Video 5. [file 41598_2024_53550_MOESM6_ESM.gif]

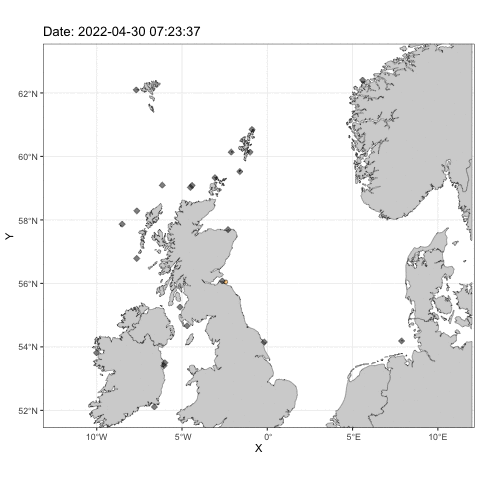

Supplement: Supplementary file 7 — Supplementary Video 6. [file 41598_2024_53550_MOESM7_ESM.gif]
